# Potential Mechanisms Linking Atherosclerosis and Increased Cardiovascular Risk in COPD: Focus On Sirtuins

**DOI:** 10.3390/ijms140612696

**Published:** 2013-06-17

**Authors:** Graziamaria Corbi, Andrea Bianco, Viviana Turchiarelli, Michele Cellurale, Federica Fatica, Aurora Daniele, Gennaro Mazzarella, Nicola Ferrara

**Affiliations:** 1 Department of Medicine and Health Sciences, University of Molise, via Giovanni Paolo II – Loc. Tappino, 86100 Campobasso, Italy; E-Mails: graziamaria.corbi@unimol.it (G.C.); andrea.bianco@unimol.it (A.B.); michele.cellurale@studenti.unimol.it (M.C.); fatifede@alice.it (F.F.); 2 Department of Scienze e Tecnologie Ambientali Biologiche Farmaceutiche, Second University of Naples, via Vivaldi 43, 81100 Caserta, Italy; E-Mail: daniele@ceinge.unina.it; 3 CEINGE Biotecnologie Avanzate Scarl, via Gaetano Salvatore 486, 80145 Naples, Italy; 4 Department of Cardiothoracic and Respiratory Sciences, Second University of Naples, via Leonardo Bianchi, Monaldi Hospital, 80131 Naples, Italy; E-Mail: gennaro.mazzarella@unina2.it; 5 Translational Medical Sciences – University of Naples “Federico II”, via Pansini 5, 80145 Naples, Italy; E-Mail: nicola.ferrara@unina.it; 6 “Salvatore Maugeri” Foundation – Institute of Telese Terme, via Bagni Vecchi 1, 82037 Telese Terme, Italy; E-Mail: nicola.ferrara@unina.it

**Keywords:** sirtuins, oxidative stress, COPD, cardiovascular diseases, atherosclerosis, inflammation, exercise training, ageing

## Abstract

The development of atherosclerosis is a multi-step process, at least in part controlled by the vascular endothelium function. Observations in humans and experimental models of atherosclerosis have identified monocyte recruitment as an early event in atherogenesis. Chronic inflammation is associated with ageing and its related diseases (e.g., atherosclerosis and chronic obstructive pulmonary disease). Recently it has been discovered that Sirtuins (NAD^+^-dependent deacetylases) represent a pivotal regulator of longevity and health. They appear to have a prominent role in vascular biology and regulate aspects of age-dependent atherosclerosis. Many studies demonstrate that SIRT1 exhibits anti-inflammatory properties *in vitro* (e.g., fatty acid-induced inflammation), *in vivo* (e.g., atherosclerosis, sustainment of normal immune function in knock-out mice) and in clinical studies (e.g., patients with chronic obstructive pulmonary disease). Because of a significant reduction of SIRT1 in rodent lungs exposed to cigarette smoke and in lungs of patients with chronic obstructive pulmonary disease (COPD), activation of SIRT1 may be a potential target for chronic obstructive pulmonary disease therapy. We review the inflammatory mechanisms involved in COPD-CVD coexistence and the potential role of SIRT1 in the regulation of these systems.

## 1. Introduction

In the last decades the worldwide population has exhibited an increasing life expectancy with a consequent rise in the elderly population, resulting in enhanced health and social costs. Ageing is accompanied by a decline in the healthy functioning of multiple organ systems, leading to an increased incidence of mortality from diseases such as type 2 diabetes mellitus, neurodegenerative diseases, and cardiovascular and respiratory disorders [1]. From a biological point of view, ageing, senescence and death represent the final steps of unsuccessful homeostasis or failure of homeodynamics [2]. The progressive increase in proinflammatory status as a major process of ageing has been defined by Franceschi as “inflammaging” [3].

Ageing-related inflammation has been associated with chronic diseases, including cardiovascular diseases (CVD), chronic obstructive pulmonary disease (COPD) and atherosclerosis [4]. Growing evidence indicates that COPD might be an independent risk factor for CVD and, at the same time, CVD patients may exhibit the coexistence of COPD [5]. The mechanisms by which CVD and COPD coexist need to be further elucidated.

The review focuses on the potential mechanisms involved in COPD-CVD coexistence and whether Sirtuins are involved in the regulation of these systems.

## 2. Endothelial Dysfunction and Inflammation

The development of atherosclerosis is a complex, multi-step process, at least in part controlled by the vascular endothelium function. It is generally believed that the state of the endothelium is influenced by several cardiovascular risk factors and by the ability of the endothelium cells to respond to different stress stimuli on the basis of a poorly defined genetic predisposition [6,7].

Endothelium is considered a critical “organ” to modulate vascular tone by sensing signals from blood and secreting active molecules [8].

Development, as well as complications of, atherosclerotic lesions, progress through structural changes in the endothelium, such as elastin degradation. Several classes of extracellular proteases participate in the breakdown of elastin and can thus influence many features of vascular remodeling in atherosclerosis [9–11]. Desmosine results from the condensation of four lysine residues between elastin proteins. Such cross-links in elastin may contribute to biomechanical properties that are essential for normal arterial function, and often deranged in CVD. In particular, elastin degradation contributes to the formation and complication of atherosclerotic lesions. Disorders of elastic fibers increase with age and contribute importantly to several non-vascular diseases, including pulmonary emphysema [9,12,13].

Under normal conditions, the endothelium is able to regulate adhesion and aggregation of leucocytes and promote fibrinolysis. Upon exposure to different triggers, endothelial cells exhibit increased permeability and decreased integrity, enhanced pro-inflammatory and reduced anti-inflammatory activity and expression of several adhesion molecules involved in cell trafficking [14].

Endothelial injury usually underlies the initial pathologic step of several pathological diseases, including atherosclerosis and CVD. Human and experimental models of atherosclerosis have identified monocyte recruitment as an early event in atherogenesis. The processes of cellular adhesion, monocyte and macrophage attachment, and transmigration of immune cells across the endothelium are crucial steps in early atherogenesis and in the later stages of mature plaque rupture [15–17], as well as in inflammatory airway cell trafficking at the epithelium level [18–20].

## 3. COPD and CVD

COPD will be the third leading cause of death by 2020 [21]. Clinical and epidemiological observations indicate that COPD patients are more susceptible to acute cardiovascular events [22–24], and about 30% die of CVD [25,26].

Some of this increase in risk is likely to come from shared factors, such as smoking [27] advanced age, decrease in physical activity, but chronic systemic inflammation could be pivotal. Indeed systemic inflammation is potentially the common pathway leading to the high prevalence of multiple chronic diseases in the same patient [28–31].

The chronicity of the inflammatory state is promoted by the production of several pro-inflammatory cytokines that increase in serum and in secretions of CVD and COPD patients [32–34]. Among them, C-reactive protein (CRP), fibrinogen, IL-1β, TNFα, MCP-1, IL-8, and IL-6 have been investigated as the most associated with progression and exacerbation of disease [35,36]. IL-β and TNFα seem to be the major stimuli that activate macrophages and transcription factors such as NF-κB, which plays a significant role in the synthesis of other pro-inflammatory cytokines and chemokines involved [37].

It has been shown that reduced lung function is associated with carotid intima-media thickness, and that atherosclerotic changes occur early in the disease process of COPD. However, the underlying mechanism for this association is still unclear [38]. Lung functional decline in COPD has also been associated with all causes of CVD mortality, independently of age, gender and baseline smoking status [38–40]. This association may be explained by common causative agents, which affect both pulmonary and cardiovascular systems [41]. There is strong evidence that systemic inflammation could explain why patients with COPD often concomitantly suffer from CVD with or without other risk factors such as arterial hypertension, hyperlipidemia and obesity [5]. Indeed, patients with low FEV1 values have high levels of inflammatory markers such as CRP and fibrinogen, which are also considered key markers of cardiac damage [42]. Mechanisms by which systemic inflammation may lead to heart failure include direct myocardial damage and accelerated atherosclerosis (plaque genesis, progression and rupture) [43].

Biomarkers associated with COPD include markers of systemic inflammation (CRP and fibrinogen) and indicators of disease activity (airways neutrophils and desmosine), which have also been related to increased vascular risk and atherogenesis [44–46]. CRP was the first biomarker to be investigated in COPD. Higher CRP levels were associated with lower lung function in 1000 New Zealanders ages 26 to 32 unrelated to smoking, obesity, and asthma [47]. Whilst Dahl *et al.* found an association between CRP levels, hospitalization and death in a population study [48], the relationship between CRP levels and mortality remains controversial.

An additional potential biomarker in COPD is fibrinogen, an acute phase plasma protein. Many cross-sectional studies have shown that blood fibrinogen levels are higher in individuals with COPD compared with healthy controls independently of current smoking status. Among both healthy individuals and COPD patients an inverse association between levels of fibrinogen and FEV1 has been reported [49]. Serum and urine levels of desmosines have also been linked to COPD pathophysiology and represent a potentially attractive indicator of elevated lung elastic fiber turnover [50].

## 4. Sirtuins and Atherosclerosis

Atherosclerosis is considered an ageing disease, and increasing age is an independent risk factor for the development of atherosclerosis [51]. Atherosclerosis is also associated with premature biological ageing. Indeed, atherosclerotic plaques show features of cellular senescence in terms of reduced cell proliferation, irreversible growth arrest and apoptosis, elevated DNA damage, epigenetic modifications, and telomere shortening and dysfunction [52]. A family of enzymes consisting of NAD^+^-dependent histone/protein deacetylases, called sirtuins, has been recently identified as the pivotal regulators of lifespan and health. In particular, overexpression of Sir2 (silencing information regulator 2), the first gene of this family discovered in yeast, has been demonstrated to extend the life span in various organisms [53], although this evidence has not been confirmed in other studies [54].

In mammals, seven sirtuins have been described, with Sirt1 being the closest homologue to the yeast Sir2 [55]. The role of sirtuins in the cardiovascular system has been investigated in both *in vitro* and *in vivo* studies In particular, SIRT1 appears to play a regulatory role in endothelial function by acting at multiple levels, including inhibition of senescence by deacetylation of p53 [56] and LKB [57,58] and regulation of angiogenic functions via deacetylation of FOXO1 [59] and NOTCH1 [60]. Sirt1 is also implicated in regulating endothelial nitric oxide and endothelium-dependent vascular tone by deacetylating nitric oxide synthase [61]. It also appears to influence genomic stability, by regulating epigenetic silencing and chromatin modification, at least in part, through direct regulation of modifying enzymes, such as the histone methyltransferase SUV39H1 [62–64]. In addition, there is evidence that SIRT1 protects against endothelial dysfunction by preventing stress-induced premature senescence, thereby modulating endothelial dysfunction in the progression of CVD [4,56,65–68]. Sirt1 also provides protection against apoptosis and plays an essential role in mediating the survival of cardiac myocytes under stress *in vitro* [68,69].

Sirtuins also appear to have a prominent role in vascular biology, and may regulate aspects of age-dependent atherosclerosis. Part of these effects may come through the regulation of lipid and cholesterol metabolism, including the ability of SIRT1 to modulate the activity of the nuclear Liver X receptor (LXR), a critical factor in reverse cholesterol transport. Li *et al.* demonstrated that SIRT1 is a positive regulator of LXR proteins and cholesterol efflux, suggesting that the interaction between diet and genetic factors could affect the progression of age-associated atherosclerosis through deacetylation of LXR by SIRT1 [70]. Moreover, a conditional deletion of SIRT1 in endothelial cells has been demonstrated to impair the angiogenic response following an ischaemic insult [59,64].

On the contrary, SIRT1 activation can inhibit vascular smooth muscle cell hypertrophy, which has been considered one of the critical contributors to atherosclerosis, and application of resveratrol prevents oxidative stress induced human coronary smooth muscle cell proliferation through inhibiting ERK activation [71,72].

SIRT1 may prevent atherothrombosis by downregulating the endothelial expression of tissue factor. Treatment of wild-type mice with the SIRT1 inhibitor splitomicin *in vivo* enhanced tissue factor activity and markedly reduced the coagulation time in a photochemical vascular injury model [73].

Interestingly SIRT1 appears to counteract atherosclerosis by the regulation of tissue metalloproteinase 3 (TIMP3), an endogenous enzyme that antagonizes vascular inflammation [74].

Although there are fewer studies of the other sirtuins, the importance of SIRT3 for cardiac function has been demonstrated by some authors. SIRT3 is expressed abundantly in the heart, and has been reported to play a protective role against hypertrophy, acting at different levels. SIRT3 overexpression blocks hypertrophy both *in vitro* and *in vivo*, whereas SIRT3−/− mice exhibit enhanced susceptibility to hypertrophy [75], though it is also likely that it indirectly protects against cardiac hypertrophy by specifically controlling ROS levels.

More recently Cardus *et al.* demonstrated that the presence of SIRT6 in endothelial cells protects from telomere and genomic DNA damage, thus preventing a decrease in replicative capacity and the onset of premature senescence. These findings suggest that SIRT1 and SIRT6 collaborate at different levels to maintain endothelial homeostasis, with SIRT6 regulating chromatin functions and DNA repair, and SIRT1 intracellular signalling networks [53].

Finally SIRT7 seems to be an essential regulator of tissue homeostasis in the heart through its interaction with p53. Sirt7-deficient primary cardiomyocytes show an approximately 200% increase in basal apoptosis, and a significantly reduced resistance to oxidative and genotoxic stress [76].

Subjects experiencing low-grade chronic inflammation diseases [77,78], including atherosclerosis and COPD, appear to benefit from physical exercise. The mechanisms by which physical inactivity interferes with chronic disease include the accumulation of visceral fat. It has been demonstrated that changes in visceral obesity degree are also related to prolonged QTc intervals modification, a major cardiovascular risk [79]; high levels of insulin have also been implicated [80], by a calcium-independent contractility modulation [81]. Insulin sensitivity, mitochondrial enzyme activity, and mixed muscle protein synthesis values in adult humans were positively affected by participation in aerobic exercise programs [82,83]. It has been observed that chronic aerobic exercise enhances muscle mitochondrial biogenesis through a calcium-regulated signaling pathway [84], and stimulates 5-AMP-activated protein kinase (AMPK) activity with subsequent increases in fatty acid oxidation and glucose uptake in skeletal muscle [85–87].

Despite the fact that such adaptations have been analyzed for several decades, the exact mechanism behind the effects of exercise on increasing mitochondrial function remains incompletely defined. Interestingly, Chen *et al.* showed that Sirt1 is required for this effect during physical activity. In particular, an up-regulation of physical activity requires the gene that codes for Sirt1. The molecular mechanism for this increase in physical activity is not known. It is possible that calorie restriction triggers changes in brain regions that govern physical activity and that Sirt1 is a regulator of this pathway [88].

SIRT1 plays a role in muscle gene expression by modulation of the cytosolic NAD^+^-to-NADH (reduced form of nicotinamide adenine dinucleotide) ratio [89]. In fact, SIRT1 forms a complex with the acetyltransferase PCAF and MyoD and, when overexpressed, retards muscle differentiation. This mechanism requires its NAD-dependent deacetylase activity. In particular, SIRT1 decreased expression of myogenin, MEF2C, genes coding for muscle structural proteins and other gene products up-regulated during muscle differentiation [89]. Since the cytosolic NAD^+^-to-NADH ratio changes during muscle contraction [90], it is possible that SIRT1 contributes to skeletal muscle adaptations with endurance exercise. Recently Koltai *et al.* found that in skeletal muscle of both young and old rats, exercise training reverses the decreases in nicotinamide phosphoribosyltransferase (NAMPT) and NAD content that occur during aging; this is thought to be mediated by increased SIRT1 activity [91].

Moreover, it has been demonstrated that prolonged moderate exercise training is able to reduce oxidative stress levels [92] and to induce an increase in SIRT1 activity in the heart and adipose tissue of aged rats, suggesting that chronic exercise, by inducing SIRT1 activity, exerts an antioxidant effect [93]. Because it is known that exercise training exerts its beneficial effects particularly on the cardiovascular system Ferrara *et al.* [93] tested FOXO3a and its targets involvement in the heart of aged trained rats, showing that exercise training enhanced FOXO3a protein expression. This was associated with a decrease in cyclin D2 and an increase in GADD45a mRNAs in the hearts of aged rats. In adipose tissue, an increase in FOXO3a protein expression and a decrease in cyclin D2, but no changes in GADD45a mRNAs, were found. This is probably in relation to higher oxidative stress in this tissue that would induce the adipocytes to choose apoptosis or necrosis rather than repair as a mechanism of detoxification [93].

Both type and volume of exercise training appear to affect exercise-induced SIRT1 activity, and therefore, the antioxidant systems. Conti *et al.* investigated the effects of chronic exercise training on antioxidant enzymes activities and oxidants sera levels in three different groups of athletes (aerobic, anaerobic and mixed sport). In addition the authors also explored *in vitro* the effects of serum from the three exercise groups demonstrating that survival and proliferation rates; SIRT1 activity after stress induction was higher in endothelial cells supplemented with the serum of athletes training in aerobic exercise than in anaerobic or mixed sport [94,95]. Subsequently it has been demonstrated that small changes in exercise volume might strongly influence training effects and particularly that the benefits of aerobic exercise could be abrogated when the same training is performed at a greater load [96,97].

## 5. Sirtuins and COPD

Many studies demonstrate that SIRT1 exhibits anti-inflammatory properties *in vitro* and *in vivo* as well as in clinical studies (e.g., patients with COPD) [37,73,98–104].

SIRT1 has recently been shown to be reduced in lung cells from COPD patients as a result of post-translational oxidative modification by cigarette smoke derived components, leading to increased acetylation and enhanced inflammatory responses to cigarette smoke [103]. Thus SIRT1 may have an important role in the regulation of inflammatory processes involved in the pathogenesis of COPD [105].

Lung cellular senescence is accelerated in COPD, which has been found to be independently associated with lowered antioxidant defense, elevated oxidative stress, protease/antiprotease imbalance, and elastolysis [106]. The telomere length in circulating lymphocytes is shortened (*i.e.*, replicative senescence) in patients with COPD compared to non-smokers [107–110]. Furthermore, the telomere length was positively correlated with PaO2, SaO2, 6-minute walking distance, and lung function in patients with COPD [109,110].

SIRT1 is shown to regulate inflammation, senescence, autophagy/apoptosis, and ageing by deacetylating histones/nonhistone proteins including transcription factors, coactivators, and other signaling molecules, such as FOXO, HIF-2a, p53 and NF-κB. The NF-κB pathway is pivotal in the pathogenesis/development of COPD by increasing release of proinflammatory mediators leading to chronic inflammation in the lung. Indeed, NF-κB in lung epithelium functions as a promoter by inducing the influx of inflammatory cells. The anti-inflammatory property of SIRT1 is associated with decreased NF-κB transcriptional activity [111].

Because of a significant reduction of SIRT1 in rodent lungs exposed to cigarette smoke and in lungs of patients with COPD [103,111], activation of SIRT1 may be a potential target for COPD therapy. Indeed, inhibition of SIRT1 enhanced NF-κB activation, whereas up-regulation of SIRT1 and resveratrol attenuated proinflammatory mediators release in response to cigarette smoke exposure [103]. However, it needs to be further understood whether SIRT1 activators protect lung against cigarette smoke-induced immune-inflammation, tissue injury, senescence and endothelial dysfunction (acetylation of eNOS, adiponection, and caveolins). It is likely that SIRT1 activation reduces lung inflammaging by down-regulating oxidative stress-mediated cellular senescence [112].

Considerable recent data also support the involvement of adipose tissue in lung inflammatory processes [113]. Adipose is responsible of a wide variety of hormonal, inflammatory and metabolic interactions with other organs [81,114,115] and can serve as a factory for manufacturing bioactive molecules (adipocytokines), including proinflammatory cytokines [116].

Adipose tissue produces a number of adipocytokines, among the most abundant of which is adiponectin [116,117–119]. Recently Okamoto *et al.* [120], demonstrated a role for adiponectin as an endogenous anti-inflammatory mediator involved in both adaptive and innate immunity, by showing that adiponectin reduces the production of CXCR3 chemokine ligands by human macrophages; suggesting the existence of a new mechanism by which adiponectin may mitigate inflammation during atherogenesis by modulating adaptive immunity [120]. High levels of total Acpr30 have been reported in COPD patients suggesting that this adipokine is correlated to the serious metabolic and inflammatory complications often associated with this disease [121,122].

Daniele et al. have demonstrated that the oligomerization pattern of adiponectin is altered in COPD; in particular the higher levels of adiponectin are associated with a specific increase of high molecular weight adiponectin, the most biologically active isoform [123]. In addition, mRNA and protein levels of AdipoR1 and AdipoR2 in lung tissues from COPD demonstrate a higher AdipoR1 expression compared to AdipoR2, suggesting a specific signalling pathway of adiponectin in this disease [123].

Recently, Nigro *et al*. confirmed the direct protective role of Acrp30 in lung epithelial A549 cell lines; anti-proliferative and anti-inflammatory effects of Acrp30 through the NF-κB-AdipoR1 pathway and by up-regulation of IL-10 cytokine were demonstrated [124].

SIRT1 activity in C2C12 cells appears to be under the control of adiponectin through Ca^2+^ signaling and changes of the NAD^+^/NADH ratio [125]. The role of sirtuin activators has been explored in several clinical conditions. Donnelly *et al.* demonstrated that resveratrol inhibited inflammatory mediator release from human airway epithelial cells, as observed in inflammatory lung diseases including COPD and asthma [126]. Moreover, resveratrol, probably by activating the SIRT1 signaling pathway, inhibits the oxidative-stress-dependent phenotypical shift of primary endothelial cells induced by pro-inflammatory factors *in vitro* [127].Further studies are required to investigate the role of resveratrol on inhibition of inflammation and cellular senescence by involving SIRT1 in lung cells, representing a promising therapeutic intervention for COPD [4].

Another sirtuin (SIRT6), also associated with physiological senescence in mammals [128], has been implicated in accelerated lung aging in COPD [129].

In particular, Minaguava *et al.* demonstrated that SIRT6 had a negative impact on TGF-β-induced senescence of human bronchial epithelial cells [130]. This anti-aging SIRT6 capacity was mainly mediated by post-transcriptional proteasomal degradation of p21/waf1 (a protein which is a regulator of cell cycle progression) [131]. It is also believed that SIRT6 is reduced in COPD lung [106]. Therefore, the development of SIRT6 activators might be a potential tool for the inhibition of accelerated lung aging in COPD.

## 6. Conclusions

Growing clinical-epidemiological evidence indicates that COPD might be an independent risk factor for CVD, which, in turn, is a leading cause of death in patients with COPD. The mechanisms potentially involved in linking atherosclerosis with increased cardiovascular risk in COPD patients have not been systematically investigated. Many markers have been linked to these conditions, the majority represented by inflammatory molecules (e.g., CRP, IL-6, IL-8, TNFa and fibrinogen).

It remains unclear whether systemic inflammation in COPD is the result of systemic diffusion of the local inflammation, or is attributable to some comorbid conditions that affect the lungs. Evidence suggests that systemic low-grade chronic inflammation and oxidative stress contributes to the development of atherosclerosis and COPD.

It has been demonstrated that SIRT1 exhibits anti-inflammatory properties both *in vitro* and *in vivo,* as well as in clinical studies, by induction of antioxidant responses. SIRT1 exerts its action activating and deactivating factors such as NFκB, p53, p73, and SOD, suggesting that SIRT1 activation may be a promising strategy for treating chronic inflammatory diseases, such as atherosclerosis and COPD.

**Figure 1:**
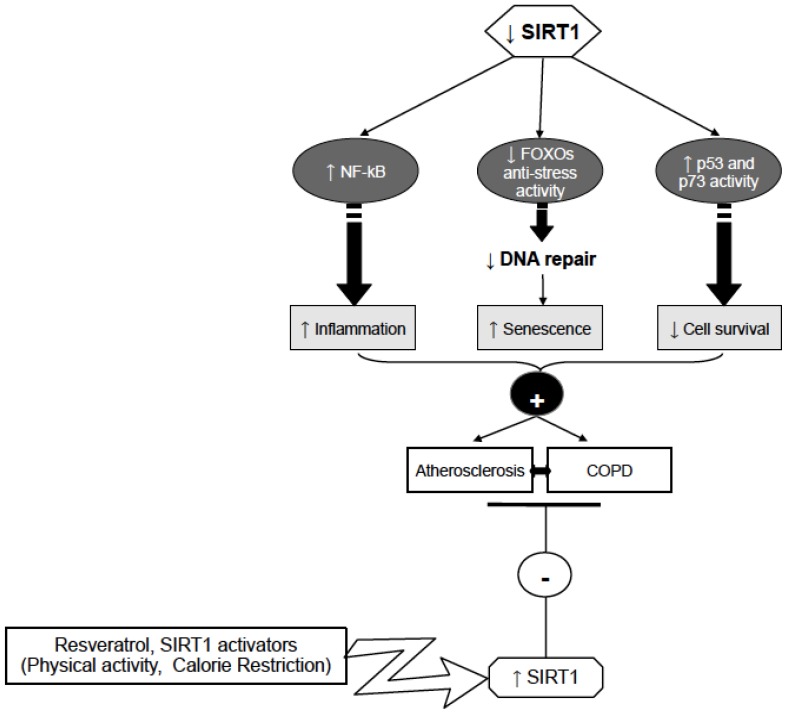
Possible mechanisms involving SIRT1 in Atherosclerosis, and COPD control. Sirt1 activation by activators such as physical activity could prevent atherosclerosis and COPD development through reduction of inflammation (by decreasing NF-κB), and increasing DNA repair (by FOXOs activation) and cell survival (by p53 and p73 activity decrease).

In light of the recent findings showing that sirtuins reduce thrombosis and inflammation, activation of this family of proteins represents a potential target for pharmacological intervention in patients with coexistent cardiovascular and lung diseases (Figure 1).

Further investigations are required to understand whether sirtuin activation is effective for treating chronic inflammatory diseases, such as atherosclerosis and COPD.
